# Supplementary material for: The hVps34‐SGK3 pathway alleviates sustained PI3K/Akt inhibition by stimulating mTORC1 and tumour growth
Source: EMBO J. 2016 Aug 1;35(17):1902–22. doi: 10.15252/embj.201693929 (PMC5007552; doi:10.15252/embj.201693929)
Supplement: Supplementary file 1 — Appendix [file EMBJ-35-1902-s001.pdf]

Appendix Data

The hVps34-SGK3 signalling module counteracts inhibition of the PI3K-Akt pathway to maintain mTORC1 activity and tumour growth

Ruzica Bago, Eeva Sommer, Pau Castel, Claire Crafter, Fiona P. Bailey, Natalia Shpiro, José Baselga, Darren Cross, Patrick A. Eyers and Dario R. Alessi

Table of Contents:

1. **Appendix Tables**
   Appendix Figure S1 (Page 2)
   Appendix Figure S2 (Page 3)
   Appendix Figure S3 (Page 4)
   Appendix Figure S4 (Page 5)
   Appendix Table S1. Sanofi 14g (Page 6)
   Appendix Table S2. Sanofi 14h (Page 7)
   Appendix Table S3. Sanofi 14i (Page 8)
   Appendix Table S4. Sanofi 14n (Page 9)

2. **Appendix Tables legend**
   General Legend applicable for Table S1 to S4 (Page 10)

A

14g

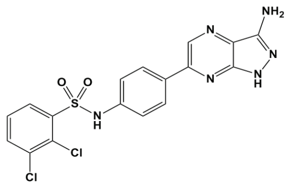

14i

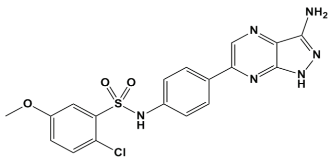

14n

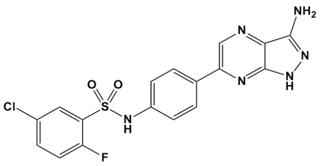

B.

kinase	14g	14i	14n
SGK1	0.05	0.07	0.013
SGK3	0.08	0.03	0.013
MLK1	0.38	0.6	0.169
MLK3	0.14	0.27	0.094
PKBa	>10	>10	>10
S6K1	0.06	0.18	0.076

Appendix Figure S1. Structure and IC50 data for 14g, 14i and 14n SGK inhibitors. (A) Chemical structures of Sanofi-14g, -14i and -14n compounds. (B) IC50 values of inhibitors on indicated recombinant kinases.

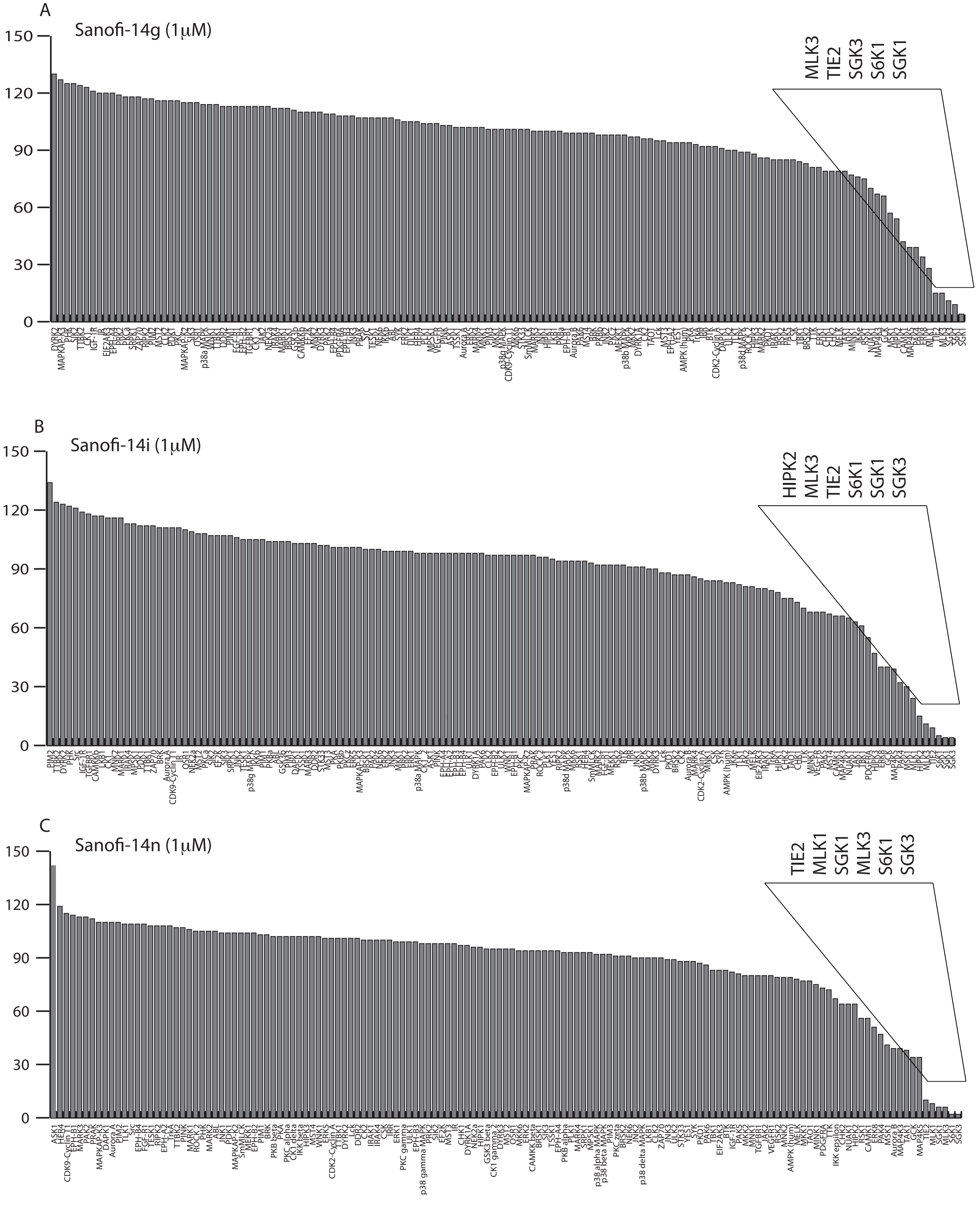

Appendix Figure S2. Protein kinase profiling of 14g, 14i and 14n SGK inhibitors. Profiling of Sanofi 14g (A), 14i (B) and 14n (C) compounds was undertaken against the Dundee panel of 140 protein kinases in the presence of 1 μM compound at the International Centre for protein Kinase Profiling. The result for each kinase is presented as a mean kinase activity of the reaction taken in triplicate relative to a control reaction where the inhibitors were omitted. Abbreviations and assay conditions are described at http://www.kinase-screen.mrc.ac.uk.

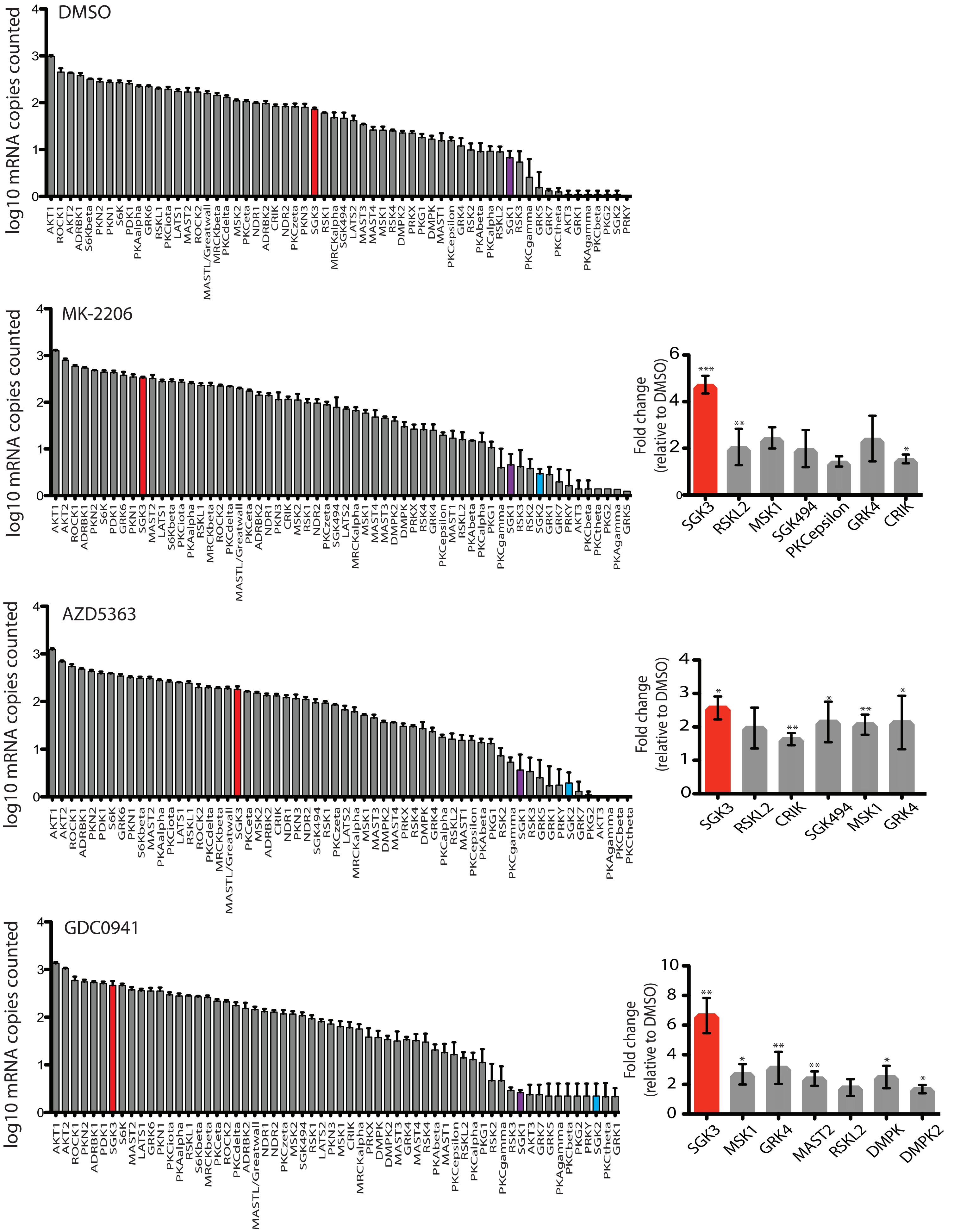

Appendix Figure S3. Human AGC kinase mRNA expression profiles in cells exposed to compounds for 2 days.
ZR-75-1 cells were grown in the presence or absence of DMSO, 1 µM MK-2206, 1 µM AZD5363 or 1 µM GDC0941 for 2 days, and total human AGC kinase mRNA levels were quantified using NanoString software. Data are presented as log10 values of triplicate data ± SD. The rank order position of SGK3 mRNA in each condition is annotated in red (left panel). All AGC kinase mRNAs exhibiting a ≥2-fold change in expression level in the appropriate compound relative to DMSO control are quantified (right panel).
*=$p<0.05$, **=$p<0.01$, ***=$p<0.001$ (right panel).

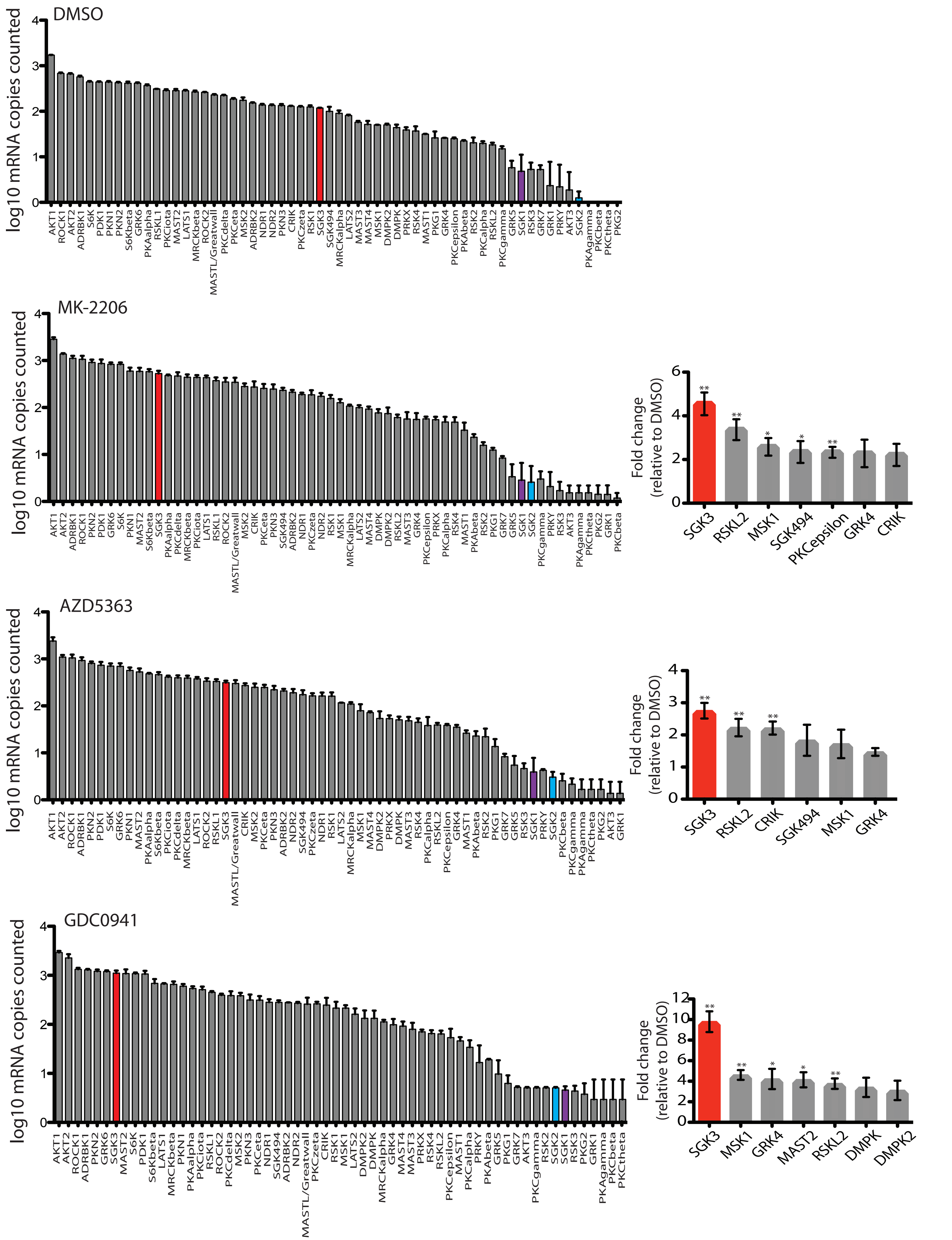

Appendix Figure S4. Human AGC kinase mRNA expression profiles in cells exposed to compounds for 5 days.
ZR-75-1 cells were grown in the presence or absence of DMSO, 1 µM MK-2206, 1 µM AZD5363 or 1 µM GDC0941 for 5 days, and total human AGC kinase mRNA levels were quantified using NanoString software. Data are presented as log10 values of triplicate data ± SD. The rank order position of SGK3 mRNA in each condition is annotated in red (left panel). All AGC kinase mRNAs exhibiting a ≥2-fold change in expression level in the appropriate compound relative to DMSO control are quantified (right panel). *=p<0.05, **=p<0.01 (right panel).

Appendix Table S1. Sanofi-14g

kinase	% activity
DYRK2	130
MAPKAP-K3	127
PHK	125
SIK2	125
TTBK2	124
CK1 delta	123
IGF-1R	121
EIF2AK3	120
EPH-A4	120
IR	120
PRK2	119
PKC alpha	118
SRPK1	118
ZAP70	118
PIM2	117
RIPK2	117
PDK1	116
PKC gamma	116
CLK2	116
MST2	116
MAPKAP-K2	115
SIK3	115
OSR1	115
p38 alpha	114
TTBK1	114
WNK1	114
CK1 gamma	113
NEK2a	113
PIM1	113
TGFBR1	113
JAK2	113
EPH-B2	113
FGF-R1	113
DDR2	113
MARK1	112
BRSK1	112
IRAK4	112
GSK3 beta	111
MNK2	110
CAMKK beta	110
DYRK3	110
EF2K	110
PAK2	109
EPH-B4	109
JNK3	108
EPH-B3	108
PDGFRA	108

kinase	% activity
PRAK	107
NEK6	107
IKK beta	107
TESK1	107
Src	107
ABL	107
SYK	106
ERK2	105
ULK1	105
HER4	105
PLK1	104
MPSK1	104
VEGFR1	104
ASK1	103
PINK	103
ERK5	102
MNK1	102
Aurora A	102
MARK4	102
TSSK1	102
MKK1	101
MKK6	101
p38 gamma	101
STK33	101
SmMLCK	101
CDK9-Cyclin	101
PIM3	101
JNK1	100
PKB alpha	100
LKB1	100
MARK3	100
HIPK1	100
Aurora B	99
PAK6	99
MST4	99
BRK	99
EPH-B1	99
JNK2	98
p38 beta	98
PKB beta	98
PKC zeta	98
MEKK1	98
MKK2	97
DYRK1A	97
CK2	96
TAO1	96
MST3	95

kinase	% activity
Lck	95
PKA	94
AMPK	94
YES1	94
EPH-A2	94
TrkA	93
CDK2-Cyclin	92
BTK	92
IRR	92
DAPK1	91
TLK1	90
ULK2	90
p38 delta	89
ROCK 2	89
HIPK3	88
PKD1	86
MARK2	86
RSK2	85
PAK5	85
IRAK1	85
CSK	85
TBK1	84
BRSK2	83
ERK1	81
TTK	81
CHK1	79
CHK2	79
MELK	79
TAK1	79
MINK1	77
IKK epsilon	76
RSK1	75
NUAK1	70
MAP4K3	67
GCK	66
MSK1	57
HIPK2	54
CAMK1	42
PAK4	39
MAP4K5	39
ERK8	34
MLK1	28
MLK3	15
TIE2	15
SGK3	11
S6K1	9
SGK1	4

kinase	% activity
MARK1	130
CSK	129
CK2	128
MAPKAP-K3	122
ASK1	121
ERK5	119
p38 alpha	117
ROCK 2	117
EPH-A2	117
Src	115
FGF-R1	115
p38 delta	114
MAPKAP-K2	114
MEKK1	114
MPSK1	114
CHK1	112
TLK1	112
PKC gamma	111
PIM1	111
TESK1	111
Lck	111
EPH-A4	111
TTBK2	110
PAK2	110
ZAP70	110
PKA	109
PHK	109
DYRK2	109
PLK1	108
SRPK1	108
IRAK4	108
PRAK	107
CDK9-Cyclin	107
PKC alpha	106
MARK4	106
PKB alpha	105
STK33	105
DAPK1	105
NEK6	105
IRR	105
DDR2	105
p38 gamma	104
MNK1	104
CDK2-Cyclin	104
BRSK1	104
MST4	104
NEK2a	103

kinase	% activity
WNK1	103
EPH-B2	103
HER4	103
JNK3	102
SIK3	102
PIM3	102
SmMLCK	101
SIK2	101
PAK6	101
SYK	101
TrkA	101
JNK2	100
CAMKK beta	100
Aurora A	100
CK1 delta	100
TTBK1	100
IKK beta	100
PIM2	100
ULK1	100
BRK	100
ERK2	99
TSSK1	99
MST2	99
OSR1	99
EPH-B3	99
EPH-B4	99
PINK	99
PKB beta	98
EF2K	98
HIPK3	98
EPH-B1	98
p38 beta	97
PKC zeta	97
MNK2	97
GSK3 beta	97
LKB1	97
BRSK2	97
IGF-1R	97
PDK1	96
AMPK	96
ERK1	95
MARK2	95
MARK3	95
RIPK2	95
YES1	95
JNK1	94
DYRK1A	94

kinase	% activity
DYRK3	94
TBK1	94
HIPK1	92
MKK6	91
MST3	91
CLK2	90
TAO1	90
IRAK1	90
ABL	90
PRK2	89
BTK	89
PDGFRA	89
PKD1	87
PAK5	87
ULK2	84
IR	82
CK1 gamma	81
EIF2AK3	81
TGFBR1	78
RSK2	77
TTK	77
JAK2	77
VEGFR1	73
MKK1	72
MINK1	71
MKK2	70
IKK epsilon	69
MELK	67
CAMK1	63
NUAK1	61
CHK2	50
RSK1	48
PAK4	46
HIPK2	45
TAK1	45
Aurora B	42
ERK8	38
GCK	34
MSK1	31
MAP4K3	25
MAP4K5	13
MLK1	10
TIE2	10
SGK1	6
MLK3	6
S6K1	3
SGK3	3

Appendix Table S3. Sanofi-14i

kinase	% activity
PIM2	134
TTBK2	124
DYRK2	123
PHK	122
Src	121
IGF-1R	119
TGFBR1	118
CAMKK beta	117
LKB1	117
MNK2	116
MARK1	116
CK1 delta	116
IRAK4	113
MPSK1	113
PDK1	112
TTBK1	112
ZAP70	112
CDK9-Cyclin	111
Aurora A	111
BRK	111
IR	111
OSR1	110
NEK2a	109
PKC alpha	108
MST2	108
SIK2	107
SIK3	107
SRPK1	107
EF2K	107
JNK2	106
p38 gamma	105
IKK beta	105
PIM1	105
TESK1	105
PKB alpha	104
GSK3 beta	104
PIM3	104
ABL	104
DAPK1	103
MARK3	103
TSSK1	103
DDR2	103
STK33	102
MST3	102
ERK5	101
PKB beta	101
PKA	101

kinase	% activity
PKC zeta	101
MAPKAP-K3	101
BRSK1	100
NEK6	100
PAK2	100
MKK1	99
ERK1	99
ERK2	99
JNK3	99
PRK2	99
p38 alpha	98
CK1 gamma	98
DYRK1A	98
PAK6	98
ASK1	98
ULK1	98
EPH-A2	98
EPH-A4	98
EPH-B3	98
EPH-B4	98
PINK	98
PKC gamma	97
MAPKAP-K2	97
PRAK	97
CLK2	97
WNK1	97
ULK2	97
EPH-B1	97
EPH-B2	97
ROCK 2	96
PLK1	96
YES1	95
MKK6	94
p38 delta	94
RSK1	94
RIPK2	94
HER4	94
SmMLCK	93
RSK2	92
MARK2	92
MEKK1	92
BTK	92
FGF-R1	92
JNK1	91
p38 beta	91
IRR	91
MKK2	90

kinase	% activity
DYRK3	90
PKD1	88
Lck	88
Aurora B	87
BRSK2	87
CK2	87
MARK4	86
CDK2-Cyclin	85
MNK1	84
CSK	84
SYK	84
AMPK	83
IKK epsilon	83
TLK1	82
MELK	81
JAK2	81
EIF2AK3	80
IRAK1	80
TrkA	79
HIPK1	78
CHK2	75
TAO1	75
CHK1	73
TTK	70
PAK5	68
MINK1	68
VEGFR1	68
MST4	67
CAMK1	66
MAP4K3	66
NUAK1	65
TAK1	63
TBK1	61
PDGFRA	55
HIPK3	47
ERK8	40
GCK	40
MAP4K5	39
PAK4	32
MSK1	30
MLK1	24
HIPK2	15
MLK3	11
TIE2	9
S6K1	5
SGK1	4
SGK3	4

kinase	% activity	kinase	% activity	kinase	% activity
ASK1	142	DDR2	101	ZAP70	90
HER4	119	CK2	100	JNK3	89
CDK9-Cyclin	115	IRAK1	100	ULK2	89
EPH-B1	114	IRAK4	100	STK33	88
MARK3	113	CSK	100	YES1	88
PAK2	113	IRR	100	SYK	88
PRAK	112	ERK1	99	PKD1	87
MAPKAP-K3	110	PKC gamma	99	PAK6	86
DAPK1	110	ULK1	99	TBK1	83
Aurora A	110	EPH-B3	99	EIF2AK3	83
PIM2	110	p38 gamma	98	BTK	83
TLK1	109	PRK2	98	IGF-1R	82
Src	109	SIK2	98	PAK5	81
EPH-B4	109	EF2K	98	MKK2	80
FGF-R1	109	MST3	98	MNK1	80
TESK1	108	IR	98	TGFBR1	80
RIPK2	108	CHK1	97	JAK2	80
EPH-A2	108	DYRK1A	97	VEGFR1	80
TrkA	108	NEK2a	96	RSK2	79
TTBK2	107	HIPK1	96	MNK2	79
PINK	107	GSK3 beta	95	AMPK	79
MARK1	106	CK1 gamma	95	MELK	78
ROCK 2	105	DYRK3	95	MKK1	77
PHK	105	MST2	95	TAO1	77
MARK2	105	OSR1	95	MINK1	75
ABL	105	MKK6	94	PDGFRA	73
JNK1	104	ERK2	94	TTK	72
PDK1	104	CAMKK beta	94	IKK epsilon	67
MAPKAP-K2	104	BRSK1	94	CHK2	64
SmMLCK	104	SIK3	94	NUAK1	64
MEKK1	104	TSSK1	94	HIPK2	64
EPH-B2	104	EPH-A4	94	RSK1	56
PIM1	103	PKB alpha	93	CAMK1	56
BRK	103	PLK1	93	ERK8	51
PKB beta	102	MARK4	93	PAK4	47
PKA	102	SRPK1	93	MSK1	41
PKC alpha	102	MPSK1	93	Aurora B	39
CK1 delta	102	p38 alpha	92	MAP4K3	39
IKK beta	102	p38 beta	92	TAK1	38
HIPK3	102	PIM3	92	GCK	34
MST4	102	PKC zeta	91	MAP4K5	34
WNK1	102	BRSK2	91	TIE2	10
ERK5	101	NEK6	91	MLK1	8
CDK2-Cyclin	101	JNK2	90	SGK1	6
TTBK1	101	p38 delta	90	MLK3	6
DYRK2	101	LKB1	90	S6K1	2
Lck	101	CLK2	90	SGK3	2

Appendix Table legend

General Legend applicable for Table S1 to S4 (Pages 6 to 9)

Results are presented as the percentage of kinase activity in DMSO control reactions. Protein kinases were assayed *in vitro* in the presence of 1µM Sanofi-14g (Table 1), -14h (Table 2), -14i (Table 3) and -14n (Table 4) compounds, as described on the International Centre for Kinase Profiling website (http://www.kinase-screen.mrc.ac.uk/), and the results are means ± S.D. for triplicate reactions. Abbreviations are as follows: ABL, Abelson tyrosine-protein kinase 1; AMPK, AMP-activated protein kinase; ASK, apoptosis signal-regulating kinase; BRK, breast tumour kinase; BRSK, brain-specific kinase; BTK, Bruton's tyrosine kinase; CaMK, calmodulin-dependent kinase; CaMKK, CaMK kinase; CDK, cyclin-dependent kinase; CHK, checkpoint kinase; CK, casein kinase; CLK, CDC-like kinase; CSK, C-terminal Src kinase; DAPK, death-associated protein kinase; DDR, discoidin domain receptor; DYRK, dual-specificity tyrosine-phosphorylated and regulated kinase; EF2K, elongation-factor-2 kinase; EIF2AK, eukaryotic translation initiation factor 2-alpha kinase; EPH, ephrin; ERK, extracellular signal-regulated kinase; FGF-R, fibroblast growth factor receptor; GCK, germinal centre kinase; GSK, glycogen synthase kinase; HER, human epidermal growth factor receptor; HIPK, homeodomain-interacting protein kinase; IGF1R, IGF1 receptor; IKK, inhibitory κB kinase; IR, insulin receptor; IRAK, interleukin-1 receptor-associated kinase; IRR, insulin-related receptor; JAK, Janus kinase; JNK, c-Jun N-terminal kinase; Lck, lymphocyte cell-specific protein tyrosine kinase; LKB1, liver kinase B1; MAPK, mitogen-activated protein kinase; MAPKAP-K, MAPK-activated protein kinase; MARK, microtubule-affinity regulating kinase; MEKK, MAP kinase kinase kinase; MELK, maternal embryonic leucinezipper kinase; MINK, misshapen/NIK-related kinase; MKK, MAPK kinase; MLK, mixed lineage kinase; MNK, MAPK-integrating protein kinase; MPSK, myristoylated and palmitoylated serine/threonine-protein kinase; MSK, mitogen- and stress-activated protein kinase; MST, mammalian homologue Ste20-like kinase; NEK, NIMA (never in mitosis in *Aspergillus nidulans*)-related kinase; NUAK, novel (NUA) family SnF1-like kinase; OSR, oxidative stress-responsive kinase; PAK, p21-activated protein kinase; PDGFRA, platelet-derived growth factor receptor-α; PDK, phosphoinositide-dependent kinase; PHK, phosphorylase kinase; PIM, provirus integration site for Moloney murine leukaemia virus; PINK (insect homologue), PTEN-induced kinase; PKA, cAMP-dependent protein kinase; PKB, protein kinase B; PKC, protein kinase C; PKD, protein kinase D; PLK, polo-like kinase; PRAK, p38-regulated activated kinase; PRK, protein kinase C-related kinase; RIPK, receptor-interacting protein kinase; ROCK, Rho-dependent protein kinase; RSK, ribosomal S6 kinase; S6K1, p70 ribosomal S6 kinase; SGK, serum- and glucocorticoid-induced protein kinase; SIK, salt-induced kinase; smMLCK, smooth muscle myosin light-chain kinase; SRPK, serine/arginine protein kinase; STK, serine/threonine kinase; SYK, spleen tyrosine kinase; TAK, TGFβ-activated kinase; TAO, thousand and one amino acid; TBK1, TANK-binding kinase 1; TESK, testis-specific protein kinase; TGFBR, TGFβ receptor; TIE, tyrosine-protein kinase receptor; TLK, tousled-like kinase; TrkA, tropomyocin receptor kinase; TSSK, testis-specific serine/threonine-protein kinase; TTBK, tau-tubulin kinase; ULK, Unc-51-like kinase; VEGFR, vascular endothelial growth factor receptor; WNK, with no lysine; YES1, Yamaguchi sarcoma viral oncogene homologue 1; ZAP, ζ-chain-associated protein.
