# Supplementary material for: The hVps34‐SGK3 pathway alleviates sustained PI3K/Akt inhibition by stimulating mTORC1 and tumour growth
Source: EMBO J. 2016 Aug 1;35(17):1902–22. doi: 10.15252/embj.201693929 (PMC5007552; doi:10.15252/embj.201693929)
Supplement: Supplementary file 2 — Expanded View Figures PDF [file EMBJ-35-1902-s002.pdf]

Expanded View Figures

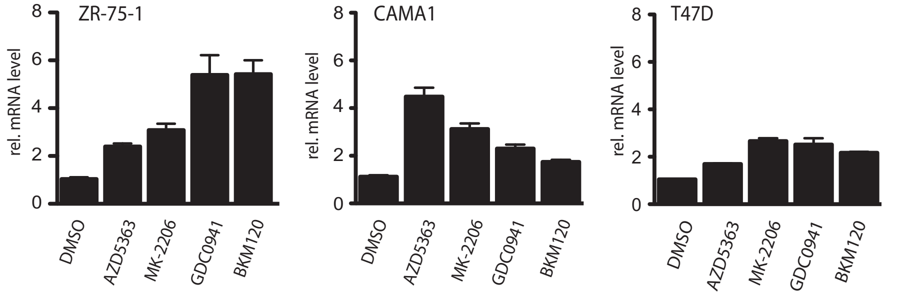

Figure EV1. Prolonged treatment with Class I PI3K and Akt inhibitors leads to upregulation of SGK3 mRNA.

ZR-75-1 (left panel), CAMA-1 (middle panel) and T47D (right panel) cells were treated for 5 days with either 1 μM MK-2206, 1 μM AZD5363, 1 μM GDC0941 or 1 μM BKM120. mRNA isolation was followed by cDNA preparation. Real-time PCR was performed on cDNA samples using specific primers against SGK3 isoform. Relative mRNA levels were calculated using $2^{(-\Delta\Delta)}$ *Ct* method using DMSO-treated samples as calibrator. Results are presented as relative mRNA level means ± SD for triplicates.

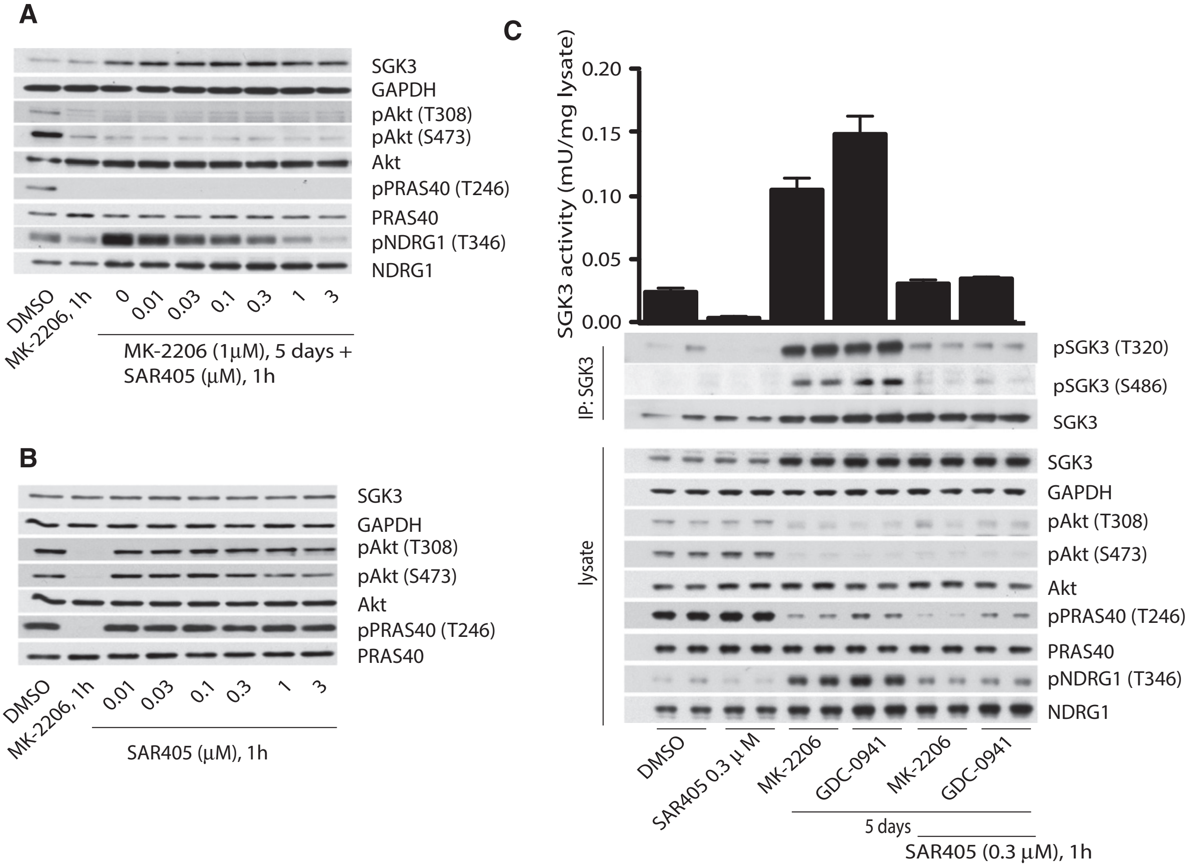

Figure EV2. Further evidence that SGK3 activity induced by inhibition of PI3K/Akt is regulated by hVps34.

A ZR-75-1 cells were treated with 1 μM MK-2206 for 5 days, and then, 1 h prior to cell lysis, cells were further treated with increasing doses of SAR405.

B ZR-75-1 cells cultured in serum in the absence of Akt inhibitor were treated for 1 h with the indicated concentrations of SAR405. The cell lysates were analysed by immunoblot using the indicated antibodies.

C ZR-75-1 cells were treated for 5 days with 1 μM MK-2206 or 1 μM GDC0941. One hour prior to lysis, the cells were incubated in the presence of absence of 0.3 μM SAR405. SGK3 was immunoprecipitated from lysates and subjected to *in vitro* kinase assay by measuring phosphorylation of the Crosstide substrate peptide in the presence of 0.1 mM [γ-^{32}P]ATP in a 30 min 30°C reaction (upper panel). Kinase reactions are presented as means ± SD for triplicate reaction. Immunoprecipitates (IP) and lysates were also analysed by immunoblot with the indicated antibodies.

 © 2016 The Authors

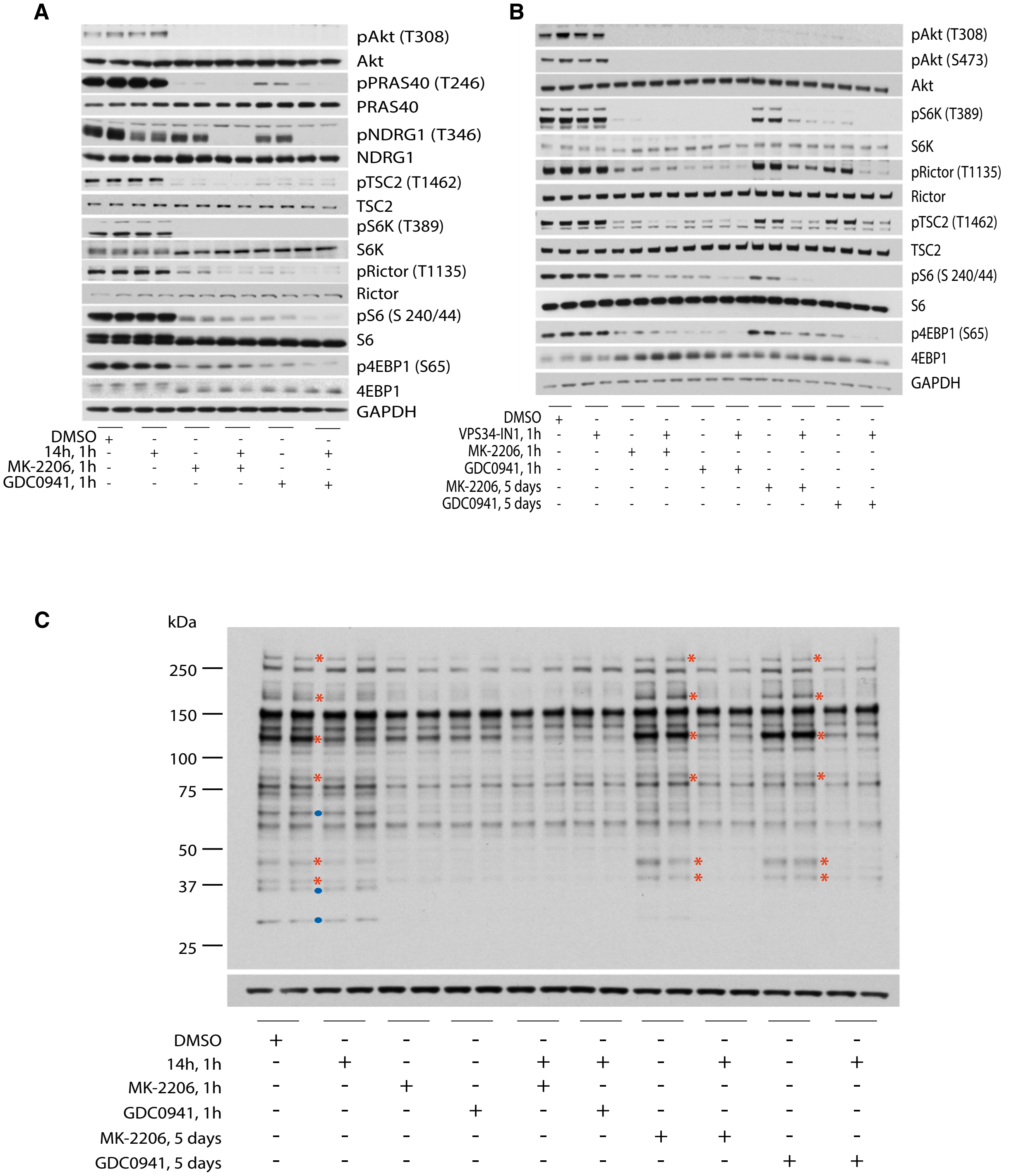

Figure EV3. Further evaluation of Class I PI3K/Akt and hVps34 inhibition on mTORC1 activity and phosphorylation of Akt substrates.

A ZR-75-1 cells were treated for 1 h with 1 μM MK-2206, 1 μM GDC0941 or 3 μM 14h inhibitors, alone or in combination, as indicated. The cell lysates were analysed by immunoblot using the indicated antibodies.

B ZR-75-1 cells were treated for 1 h or 5 days with 1 μM MK-2206, 1 μM GDC0941 or 1 μM VPS34-IN1 inhibitors, alone or in combination, as indicated. The cell lysates were analysed by immunoblot using the indicated antibodies.

C ZR-75-1 cells were treated for 1 h or 5 days with 1 μM MK-2206, 1 μM GDC0941 or 3 μM 14h inhibitors, alone or in combination, as indicated. The cell lysates were analysed by immunoblot using the antibody against phosphorylated Akt phosphorylation consensus motif (RxRxxpS/pT). Red asterisk indicates potential dual Akt and SGK3 substrates whose phosphorylation is induced following 5 day MK-2206 or GDC0941 treatment and suppressed by a subsequent 1-h treatment with 14h. Blue circles indicates potential Akt selective substrates whose phosphorylation is inhibited by MK-2206 or GDC0941 treatment and but do not become re-phosphorylated following 5-day incubation with these inhibitors under conditions where SGK3 is upregulated.

© 2016 The Authors

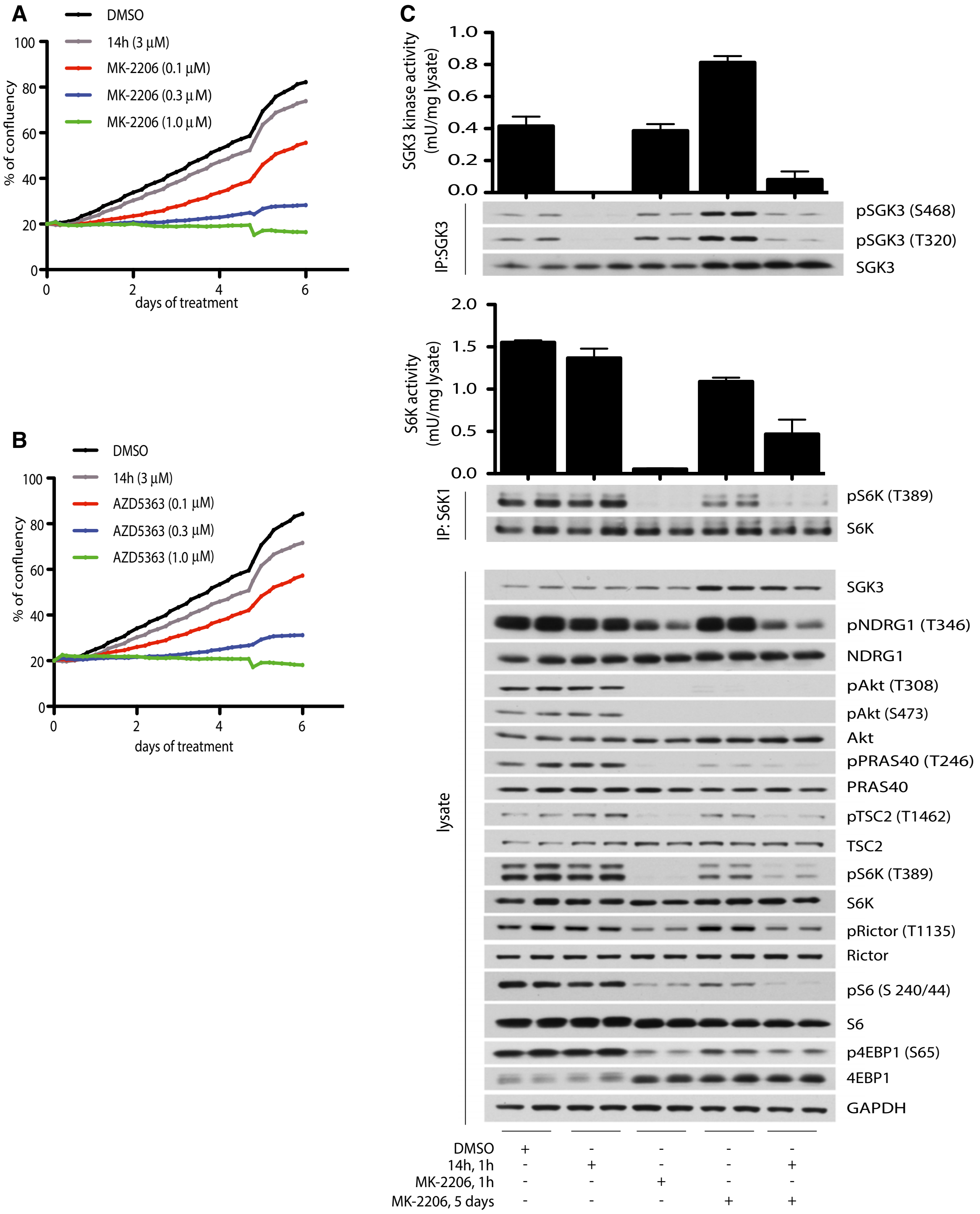

Figure EV4. Akt inhibition induces dose-dependent inhibition of cell growth in BT-474c cells *in vitro* and stimulates SGK3 to activate mTORC1.

A, B BT-474c cells were treated with DMSO, 3 μM 14h and the indicated doses of MK-2206 (A) or AZD5363 (B) inhibitors. Cell confluency was measured on the Incucyte ZOOM every 4 h for up to 6 days.

C BT-474c cells were treated for 1 h or 5 days with 1 μM MK-2206 or 3 μM 14h, as indicated. SGK3 (upper panel) and S6K1 (middle panel) were immunoprecipitated and subjected to *in vitro* kinase assay by measuring phosphorylation of the Crosstide substrate peptide in the presence of 0.1 mM [γ-^{32}P]ATP in a 30 min 30°C reaction. Kinase reactions are presented as means ± SD for triplicate reaction. Immunoprecipitates (IP) were also analysed by immunoblot with the indicated antibodies. The cell lysates were subjected to immunoblot analysis using the indicated antibodies (bottom panel).

Source data are available online for this figure.

 © 2016 The Authors
