# Supplementary figures and images for: The hVps34‐SGK3 pathway alleviates sustained PI3K/Akt inhibition by stimulating mTORC1 and tumour growth
Source: EMBO J. 2016 Aug 1;35(17):1902–22. doi: 10.15252/embj.201693929 (PMC5007552; doi:10.15252/embj.201693929)

Source Data for for Figure EV4C

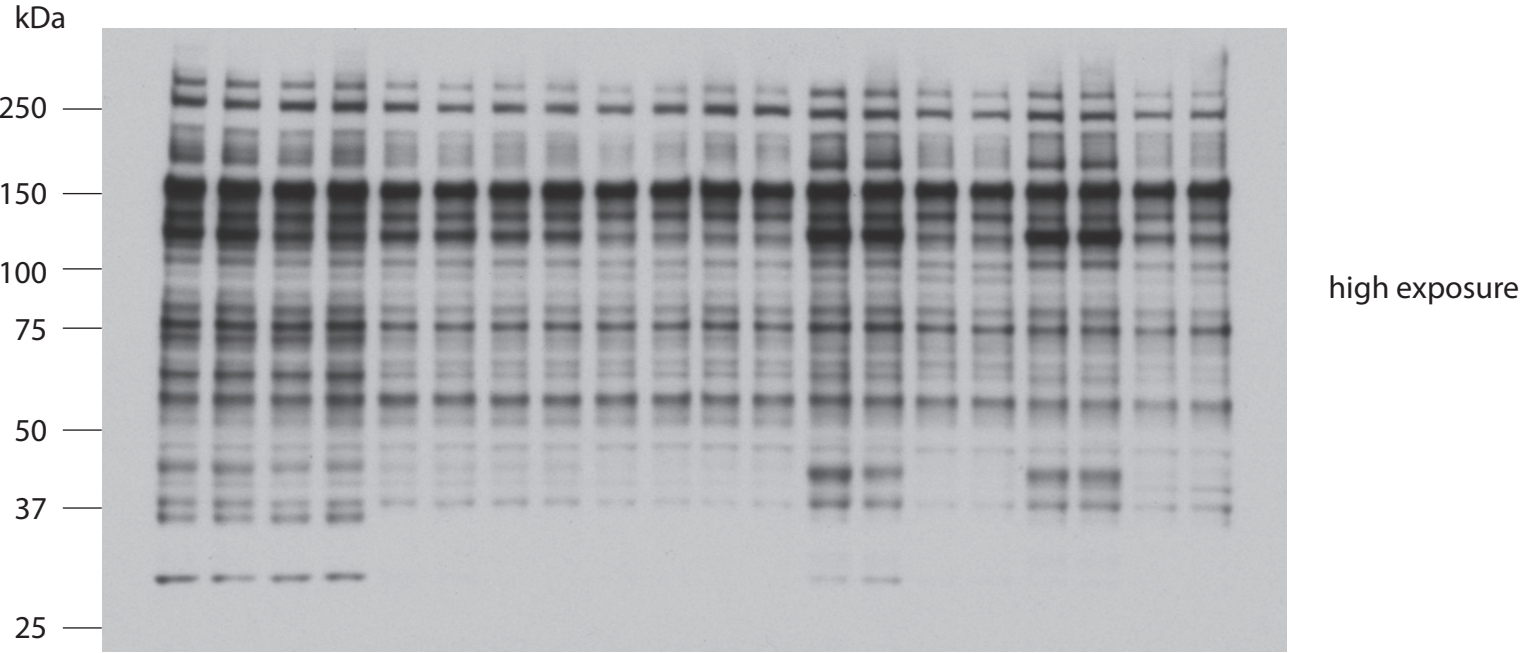

Supplement: Supplementary file 4 — Source Data for Expanded View [file EMBJ-35-1902-s005.zip › embj201693929-sup-0005-SDataEV.pdf]

Source data Figure 1C

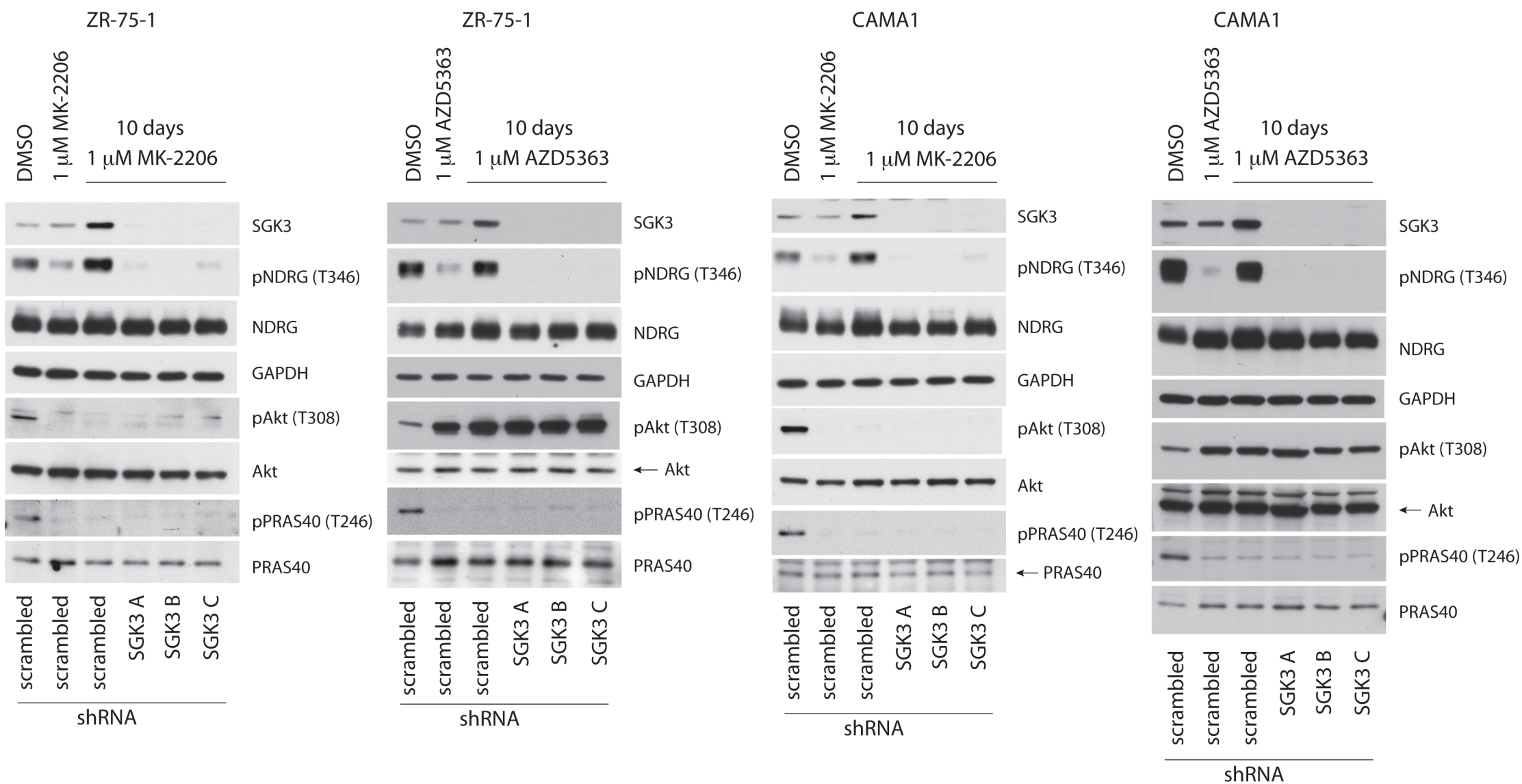

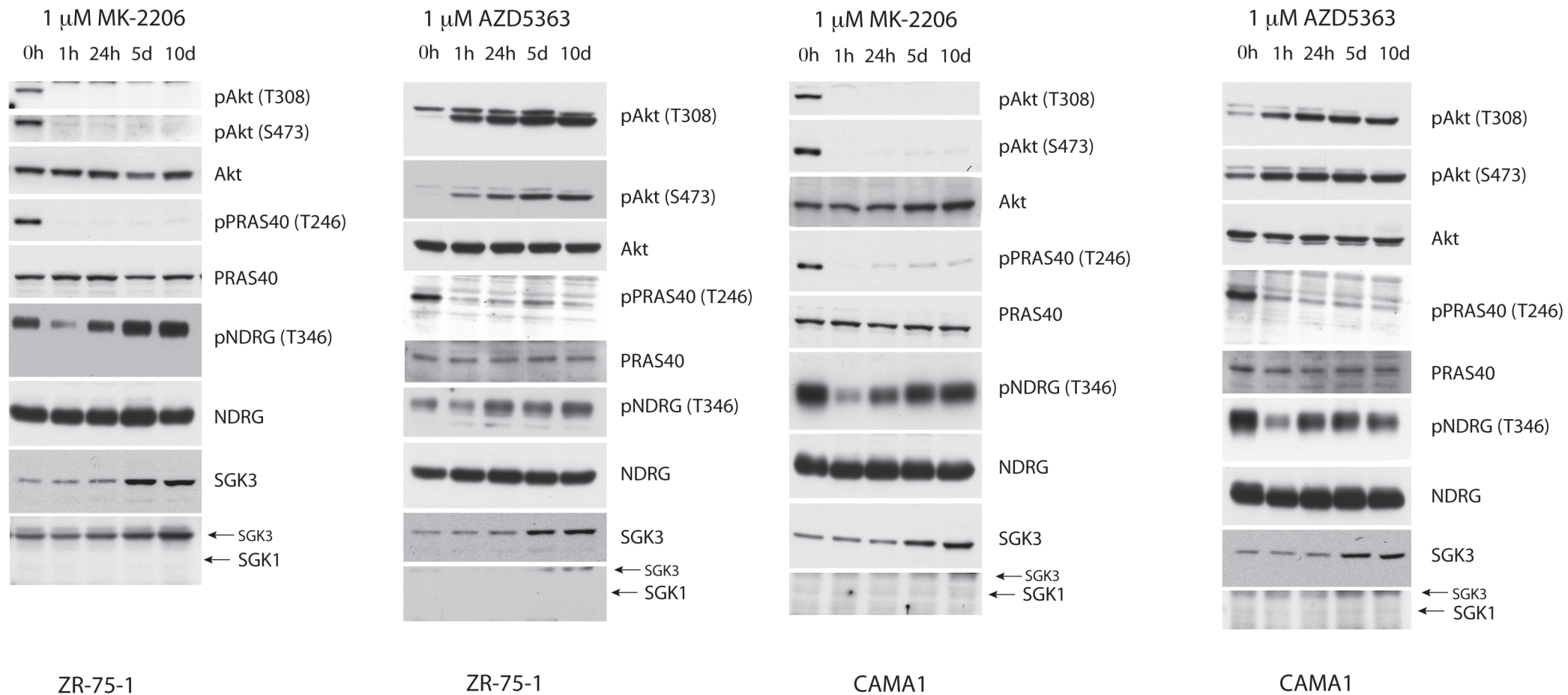

Supplement: Supplementary file 6 — Source Data for Figure 1 [file EMBJ-35-1902-s003.pdf]

# ZR-75-1

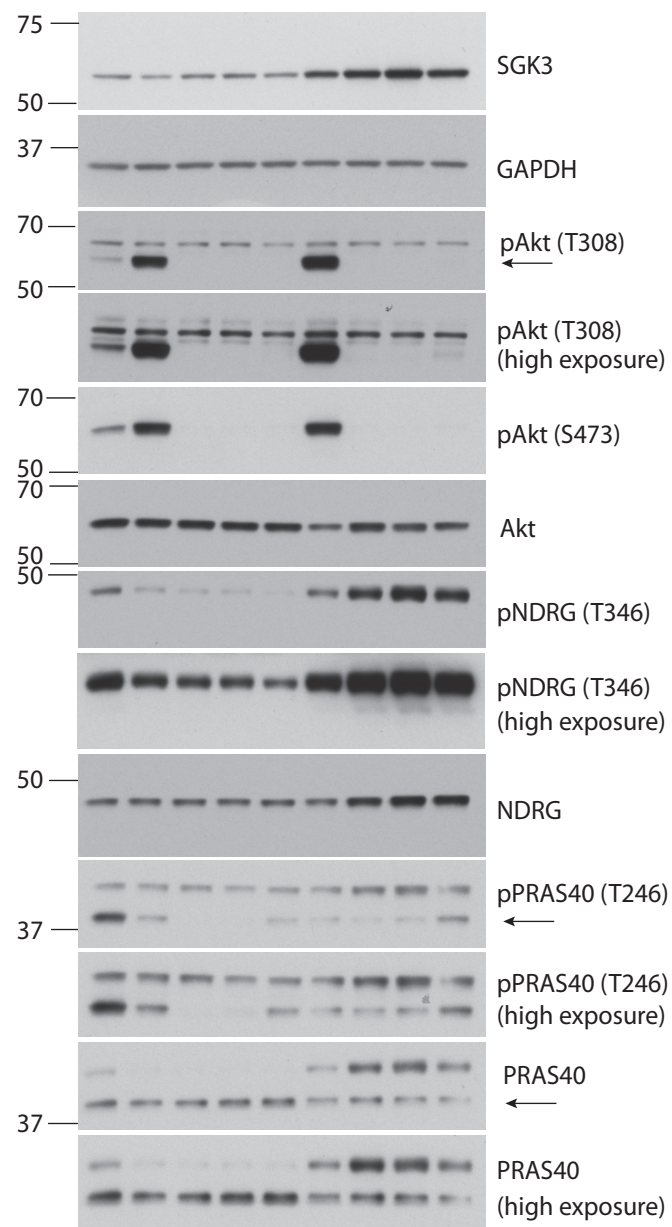

# CAMA1

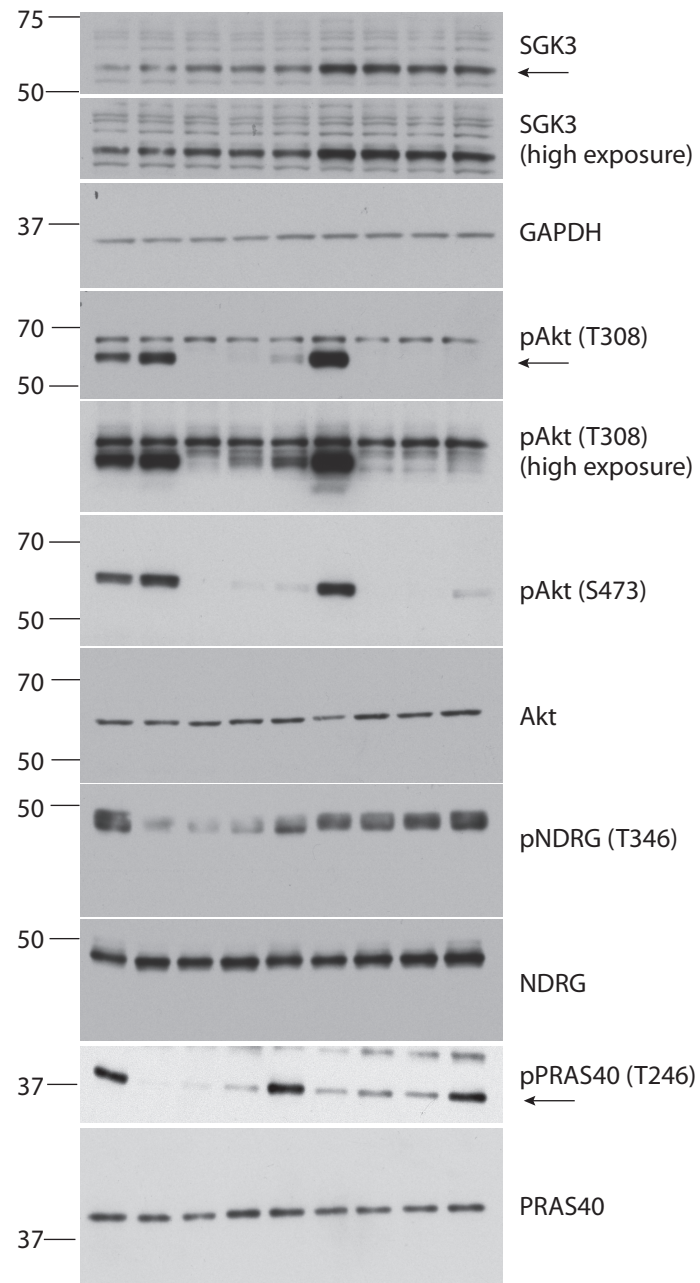

# T47D

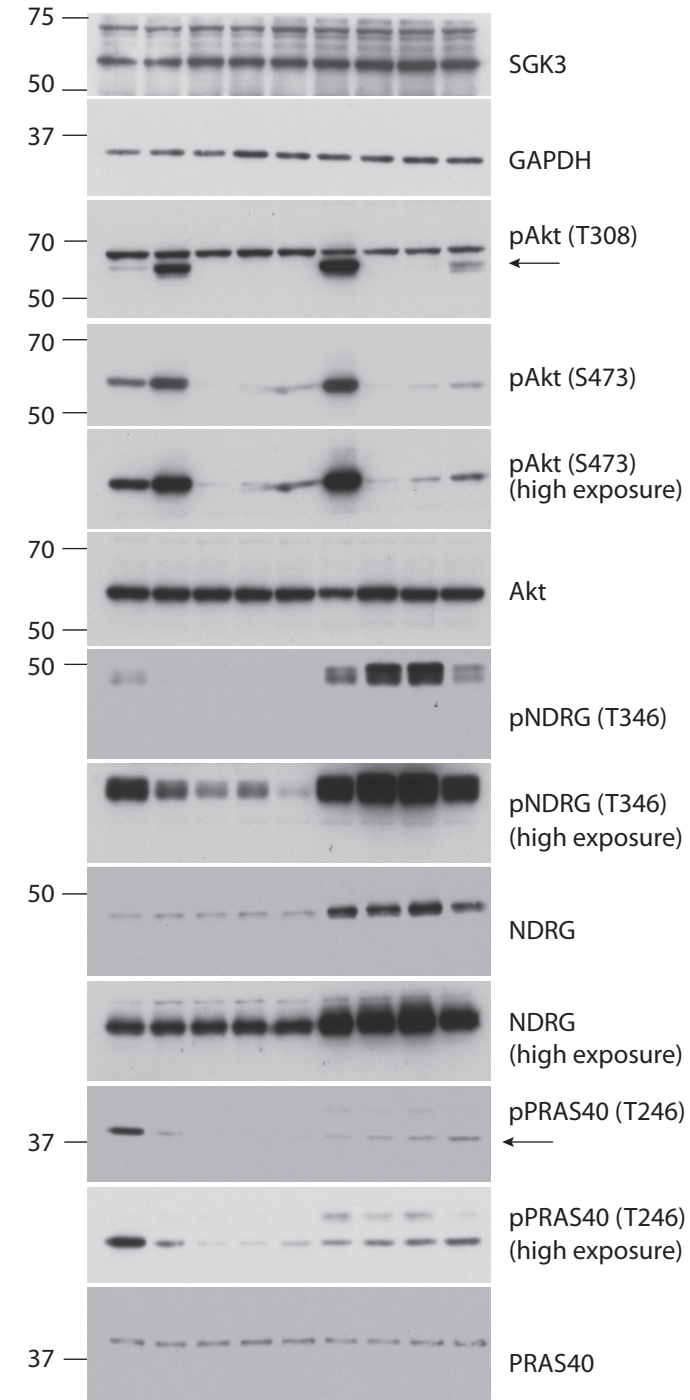

Supplement: Supplementary file 7 — Source Data for Figure 2 [file EMBJ-35-1902-s004.pdf]
